# The effect of ultrasound therapy on lateral epicondylitis: A meta-analysis

**DOI:** 10.1097/MD.0000000000028822

**Published:** 2022-02-25

**Authors:** Dongni Luo, Bingyan Liu, Lini Gao, Shengxin Fu

**Affiliations:** Department of Ultrasound, Hainan General Hospital (Hainan Affiliated Hospital of Hainan Medical University) Hainan, China.

**Keywords:** lateral epicondylitis, tennis elbow, ultrasound therapy

## Abstract

**Objective:**

Lateral epicondylitis is a common musculoskeletal disorder, and ultrasound therapy is one of the most used treatments in the clinic. The effect remains uncertain, and the present paper aims to figure it out with a meta-analysis.

**Methods:**

The Pubmed, Cochrane library, and Embase databases were searched for relevant studies published before Jure 1, 2021. Continuous variables were compared by calculating the standard difference of the means, whereas categorical dichotomous variables were assessed using relative risks. A random-effects model was used if the heterogeneity statistic was significant; otherwise, a fixed-effects model was used.

**Results:**

Thirteen studies were included in the quantitative analysis, including 442 participants (287 ultrasonic treated patients and 155 controls). The VAS scale decreased markedly after ultrasound therapy (*P* = .027). However, no statistically significant difference could be found between ultrasound therapy and the control groups at all post-treatment time points. Similarly, no benefits could be found when comparing the pre- and post-treatment grip strength with ultrasonic therapy (*P* = .324). Moreover, though ultrasound treatment always continues for a long time, the present study demonstrated there were no additional benefits when comparing short- and long-term outcomes.

**Conclusions:**

The ultrasound therapy is helpful to relieve pain for LE patients, but no such benefit could be found for grip strength. However, it has no significant advantage against other conservative treatments like rest and brace.

## 1 Introduction

Lateral epicondylitis (LE), also known as tennis elbow, is a common musculoskeletal disorder worldwide.^[1,2]^ It is caused by the tendinitis of the short radial wrist extensor tendon and results in significant pain in the elbow's lateral area.^[3]^ LE is commonly seen in elbow overuse populations like athletes and manual workers who perform repetitively, resistance-based, and wrist-extension activities. It has also been reported to have an incidence of 1% to 3% in the general population.^[4]^ LE was firstly described as early as 1873 by Runge, but the aetiology is still not well understood. The primary pathology of LE is the mechanical stimulation at the insertion area of the extensor carpi radialis brevis muscle, which results in the tendinitis status of the insertion tendon.^[5]^ Histologically, the LE tissues display a non-healing status characterized by hypercellularity, abundant proteoglycan deposition, and collagen matrix degradation without the infiltration of inflammatory cells.^[6]^

Multiple therapy methods have been developed to treat tennis elbow, including rest, brace, therapeutic exercises, pharmacology, laser, acupuncture, extracorporeal shock wave therapy, ultrasonic, and surgery.^[7]^ Though all the therapy strategies were reported useful for treating LE patients, some of the patients still suffer from pain and strength impairment after various treatments. Ultrasound therapy is a treatment modality commonly used in physical therapy, which utilizes a hand-held device to make and transport sound waves to the internal injured site.^[8]^ It provides deep heating to soft tissues in the body, including muscles, tendons, joints, and ligaments.^[8]^ The use of ultrasonic therapy in treating tennis elbow has been widely researched and used in the clinic. However, its effect remains uncertain, and many therapeutists refuse to use it.^[9,10]^ The present paper aims to figure out whether ultrasound therapy is useful for treating tennis elbow.

## 2 Method

### 2.1 Search strategy

The present study was conducted based on the Preferred Reporting Items for Systematic Reviews and Meta-analyses (PRISMA) 2020 statement. The protocol was registered at INPLASY (number: INPLASY202160073, https://inplasy.com/). The Pubmed, Cochrane, and Medline databases were searched by DNL and BYL for potential researchers published before June 1, 2021. The following search criteria were used during the literature search: (tennis elbow or lateral epicondylitis) and (ultrasound therapy or ultrasonic). Manually search was further performed for additional potential papers with the related articles function and reference screening. The language was restricted to only English.

### 2.2 Inclusion and exclusion criteria

Any study reporting the outcomes of ultrasound therapy for tennis elbow was included for further reading. All titles, abstracts, and full papers of potentially relevant studies were assessed by DNL and BYL for eligibility. Papers were included if they meet the following criteria:

1. participants were 18 years of age or older;
2. participants were diagnosed with tennis elbow for at least one month;
3. the outcomes like pain (VAS), grip strength, or functional assessment were reported;
4. Cohort study design.

The exclusion criteria were as follows:

1. Studies nor reporting the outcomes of interest;
2. Studies nor reporting the outcomes of matched time points;
3. Outcomes not reporting as mean ± SD;
4. Studies utilized other therapies like PRP injection.

When several reports from the same study were published, only the most recently or informative one was included in this meta-analysis. Disagreements were resolved through discussion and consensus, and sometimes by consultation with the corresponding author.

### 2.3 Data extraction and quality assessment

The data extraction of all baseline characteristics and outcomes of interest were performed independently by DNL and BYL. Disagreements were resolved through discussion and consensus, or by consultation with the corresponding author if needed. The methodological quality of the included studies was assessed by the Quality Index, which consisted of 27 items distributed between five sub-scales.^[11]^ Matched outcomes were checked throughout the papers. The VAS scale (pre-treatment, short-term post-treatment, mid-term outcome, and long-term outcome) and grip strength (pre-treatment, mid-term outcome, and long-term outcome) were the only matched outcomes. The two outcomes, together with other basic data like study design, year, country, and the number of participants were extracted from all the studies included. If articles reported insufficient data, we contacted corresponding authors for additional information.

### 2.4 Statistical analysis

The statistical analysis was performed using the special meta-analysis software named “Comprehensive Meta-Analysis (CMA).” Continuous variables were compared by calculating the standard difference of the means. All the results were presented as forest plots. A *P* value of less than .05 was considered statically significant, and a 95% confidence interval was given for each effect size. Heterogeneity is calculated with the *I*^2^ statistic, ranging from 0% (complete consistency) to 100% (complete inconsistency). The random-effects model was used if the heterogeneity was significant; otherwise, a fixed-effects model was used. To test the stability of the results, we conducted a sensitivity analysis by omitting each study. Finally, the risk of publication bias was assessed using Egger test, with the post-surgery VAS scale as primary outcome.

## 3 Results

### 3.1 Description of literature screening and quality assessment

**Figure 1:**
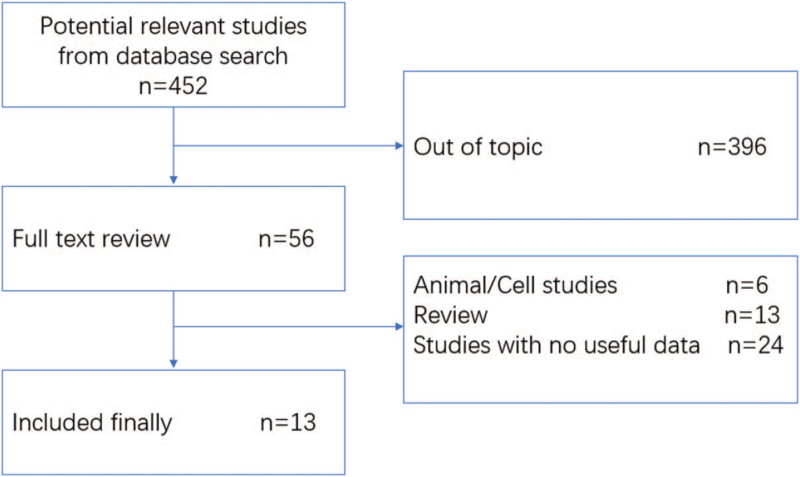
Search strategy flow diagram.

The flow diagram of the study selection process is shown in Figure 1. A total of 436 articles were obtained during the initial database screening, and additional 16 papers were retrieved through the manual search. After removing duplicates and screening the titles, 56 studies were eligible for further abstract or full-text reading. Additional 43 papers were excluded due to various reasons like lack of outcomes of interest, outcomes not presented as mean ± SD, and et al. Finally, a total of 13 studies were included in our quantitative analysis.

The characteristics of the included studies are presented in Table 1. All eligible studies were published from 1988 and were mainly conducted in Europe and North America. A total of 442 participants were included, with 287 patients treated with ultrasound (case group) and another 155 patients treated with placebo or conservative treatments (control group). All the patients were diagnosed with tennis elbow for at least one month, failed with conservative therapy. All the case group patients were treated with different dosage or course of ultrasound therapy, as shown in Table 1. The outcomes were measured at 4 timepoints, pre-treatment, short term post-treatment, mid-term post-treatment (about 6 weeks), and long-term post-treatment (about 12 weeks). Only the VAS scale and grip strength outcomes were matched for at least three studies among the included studies. On review of the data extraction, there was 100% agreement between the 2 reviewers. According to the checklist for measuring study quality, all the studies were considered high/medium-quality methodology (ranged from 17 to 23). Thus, the methodological bias of this study was considered low.

**Table 1:** The basic characteristics of the included studies.

						Number of patients			
Study	Year	Country	Inclusion criterion	Ultrasound therapy methods	Control treatment methods	Ultrasound therapy	Control	Follow-up periods	Outcomes
Panoutsopoulos et al	2020	Greece	LE	A Gymna Pulson 200 device with a model 204 transducer (4 cm^2^) was used in pulse mode, with a frequency of 3 MHz for lesions with a depth less than 2 cm, the intensity in situ was approximately 1 W/ cm^2^	Various conservative therapy	63	18	4 weeks	VAS, Functional and Quality of life Impairment
Mesci et al	2018	Turkey	Chronic LE	1 cm^2^ application area, at 1.5 W/ cm^2^, 1 MHz frequency, 5 min once a day, 5 days a week, for 10 sessions in total.	\	24		1 month	VAS, Grip strength, functional status evaluation, quality of life evaluation
Kubot et al	2017	Poland	LE	10 daily treatments, 5 cm^2^ at 0.5 W/cm^2^ and a frequency of 1 MHz.	\	30		8 weeks	VAS, Leitinen questionnaire, pain intensity and frequency,
Rahman et al	2017	Bangladesh	LE	Ultrasound therapy of 3 MHz, 1.5 W/ cm^2^, and pulsed mode of 1.5 ms on and 4 ms off.	local steroid injection, eccentric exercises and advised to avoid provocative activities	40	40	2 weeks	VAS
Best et al	2015	USA	LE	3 MHz frequency, 0.132 W/ cm^2^, intensity per applicator, continuous wave form, BNR: <5:1, ERA: 6 cm^2^ per applicator; up to 4 h permtreatment session	\	11		6 weeks	VAS, Grip strength
Murtezani et al	2015	Kosovo	LE for more than 3 months	A pulsed mode with a 20% duty cycle, intensity of 1.5 w/ cm^2^, 1MHz, for 5 to 7 min on 3 days a week for 6 weeks	local infiltration of 1 mL triamcinolone acetonide (10 mg/mL) and 1 mL lidocaine 2%; a total of 2 injections	25	24	12 weeks	VAS, PRTEE, and painfree grip strength
LIZIS et al	2015	Poland	LE	intensity, 0.8 W/ cm^2^; 100% fill; carrier frequency, 1 MHz. The patients received a series of 10 treatments 3 times per week. Each treatment session did not exceed 10 min.		25		12 weeks	Grip strength, VAS
Radpasand et al	2009	USA	Chronic LE	The ultrasound was set at 3 MHz, 1.5 W/ cm^2^, and pulsed mode of 1 ms on and 5 ms off.		2		12 weeks	PRTEE, VAS, Grip strength
Ö ken et al	2008	Turkey	LE	A frequency of 1 MHz and intensity of 1.5 W/ cm^2^ for 5 minutes in 5 days per week for 2 weeks plus a hot pack on the LE region, for ten sessions.	Bandage during the daytime for two weeks	19	20	6 weeks	Grip Strength, Pain Severity, Global Assessment of Improvement
Ostor et al	2006	United Kingdom	Chronic LE	Lowintensity (30 mW/ cm^2^), 1.5MHz ultrasound signal modulated by an ON/OFF square function	placebo	25	23	12 weeks	VAS, PRFEQ, grip strength, and a summary status of local injury questionnaire
Pienimaki et al	1998	Finland	Chronic LE	pulse 1:5, 0.5 W/cm^2^, up to 15 treatment visits	four-step progressive strengthening and stretching arm exercise	11	12		VAS
Lunceberg et al	1988	Sweden	LE for more than 1 months	frequency of 1.0 MHz, intensity of 1.0 Wcm^−2^ and was applied for 10 min, twice per week, totally 5-6 weeks.	placebo	12	10	12 weeks	VAS, Satisfactory
					rest		8		

### 3.2 Main analysis

**Figure 2:**
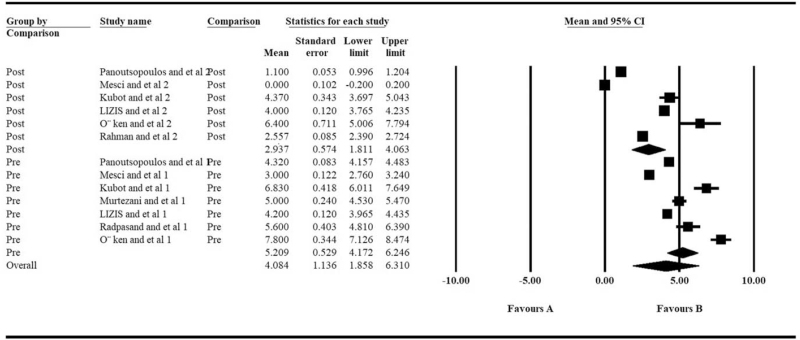
Difference of the post- vs pre- VAS scale of the ultrasound therapy group: the forest plots present each study's mean VAS score with a random effect model. Each square represents the individual study's mean score with a 95% CI indicated by the horizontal lines.

**Figure 3:**
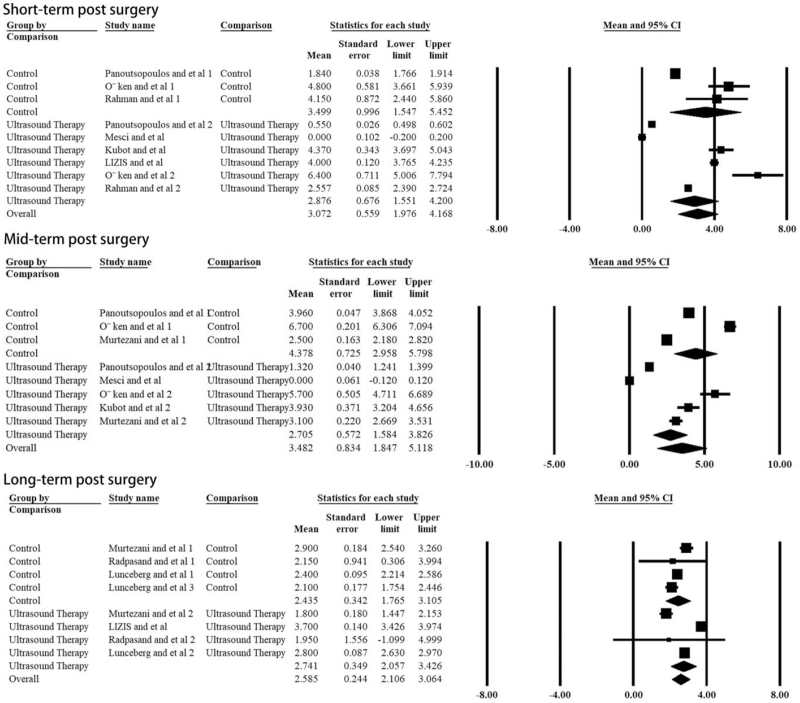
Difference of the VAS scale between ultrasound and control groups at different post-surgery time points: the forest plots present each study's mean VAS score with a random effect model. Each square represents the individual study's mean score with a 95% CI indicated by the horizontal lines.

The most significant symptom of tennis elbow is pain, so the most important outcome for evaluating the effect of ultrasound therapy is pain relief. The VAS scale comparison was available at 4 time points, pre-treatment, short-term post-treatment, mid-term post-treatment (about 6 weeks), and long-term post-treatment (about 12 weeks). There was no significant difference with the pre-treatment VAS scale between the groups (*P* = .734, Table 2). The VAS scale decreased markedly after ultrasound therapy (*P* = .004, Table 2, Fig. 2). However, no statistically significant difference could be found between ultrasound therapy and the control group at all post-treatment time points (Table 2, Fig. 3).

**Table 2:** The extracted data of matched outcomes.

	Control	Case	Effect model	*P*
VAS				
Pre	5.497	5.204	Random	.734
Short-term post	4.378	2.705	Random	.07
Mid-term post	3.707	2.558	Random	.199
Long-term post	2.435	2.741	Random	.531
Grip strength				
Pre	40.064	32.686	Random	.327
Mid-term post	38.042	37.385	Random	.86
Long-term post	37.48	38.756	Random	.583
Cases VAS				
Post vs Pre	Pre	Post	Random	.004
	2.937	5.209		
Cases VAS				
Long-term vs Short-term	Short-term post	Long-term post	Random	.884
	2.552	2.678		
Cases Grip strength				
Post vs Pre	Pre	Post	Random	.324
	38.815	33.136		
Cases Grip strength				
Long-term vs Short-term	mid-term post	Long-term post	Random	.376
	35.742	38.723		

**Figure 4:**
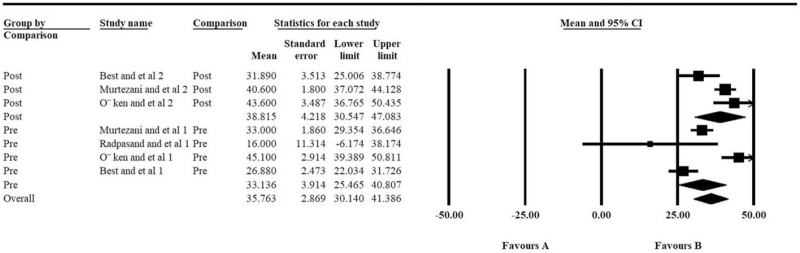
Difference of the post- vs pre- grip strength of the ultrasound therapy group: the forest plots present each study's mean grip strength with a random effect model. Each square represents the individual study's mean score with a 95% CI indicated by the horizontal lines.

**Figure 5:**
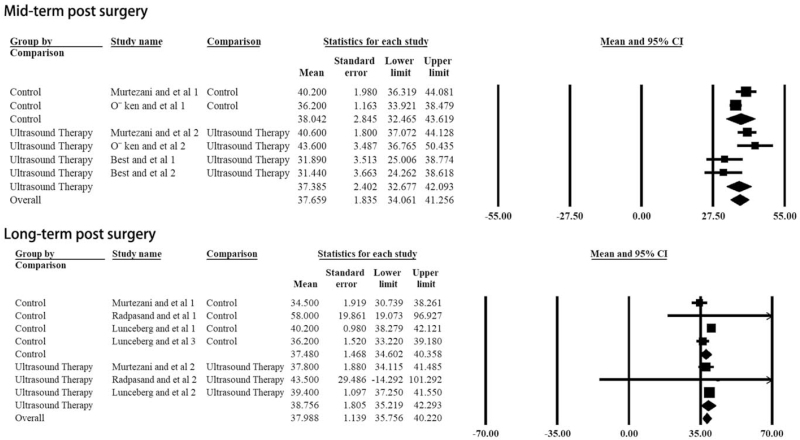
Difference of the grip strength between ultrasound and control groups at different post-surgery time points: the forest plots present each study's mean grip strength score with a random effect model. Each square represents the individual study's mean score with a 95% CI indicated by the horizontal lines.

However, for the comparison of grip strength, another important outcome for measuring treatment effectiveness, no statistical difference improvement could be found when comparing post and pre grip strength in the ultrasound group (*P* = .324, Table 2, Fig. 4). Also, there was no statistical difference between the ultrasonic and the control groups, no matter what time point was (Table 2, Fig. 5).

**Figure 6:**
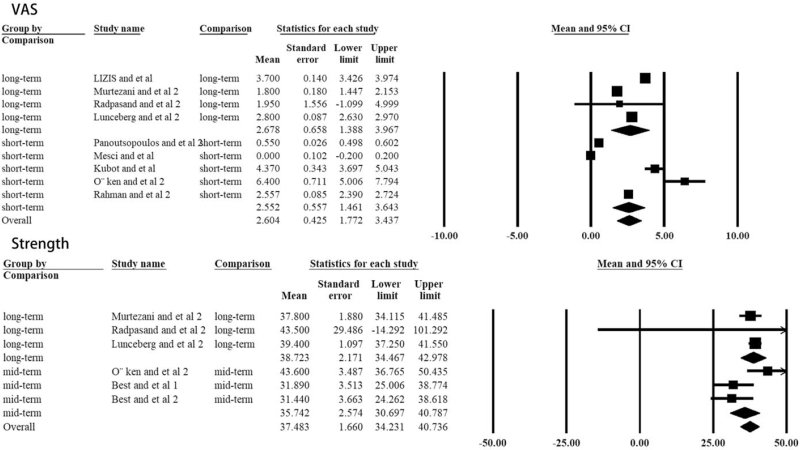
Difference of the VAS scale and grip strength in ultrasonic therapy patients at different time point. The forest plots present each study's mean VAS/grip strength with a random effect model. Each square represents the individual study's mean score with a 95% CI indicated by the horizontal lines.

Moreover, though ultrasound treatment sometimes continues for a longtime, the present study demonstrated there was no additional benefits as no statistical difference could be found between short- and long-term VAS/Strength (Table 2, Fig. 6).

### 3.3 Publication bias

No publication bias was found among the studies (*P* = .101).

## 4 Discussion

The present study demonstrated the ultrasound therapy is helpful to relieve pain for tennis elbow patients, but no such benefit could be found for grip strength. The ultrasonic treatment group also showed no advantage against other conservative treatments (like rest, brace, NSAIDs). Moreover, though ultrasound treatment sometimes continues for a longtime, the present study demonstrated no additional benefits when comparing short- and long-term outcomes. Lateral epicondylitis, which is always treated with conservative treatments including rest and exercise, NSAIDs, and local corticosteroid injection, is one of the most common musculoskeletal system disorders. Recently, ultrasound therapy has become increasingly utilized in the clinic treatment by many physiotherapists considering the good outcomes found in animal studies.^[12,13]^ However, the doubts about the effect in the clinic have not disappeared at all.^[14,15]^

Ostor et al demonstrated that low-intensity ultrasound therapy was no more effective than placebo for recalcitrant LE in a randomized, double-blind, placebo-controlled trial of 55 subjects.^[9]^ Another group also demonstrated that a brace has a shorter beneficial effect than ultrasound therapy in reducing pain.^[10]^ Similar results were found based on other types of musculoskeletal conditions like subacromial bursitis and chronic patellar tendinopathy.^[16,17]^ So, whether ultrasound therapy is helpful or is just something like a placebo is quite essential. The present study demonstrated the ultrasonic treatment helps release pain for tennis elbow, but no better than conservative treatments, and no benefits could be found with the grip strength. It means the ultrasonic therapy is better than the placebo and could be a choice for LELE management. However, if compared with conservative treatments, the application of ultrasound therapy seems dispensable when considering the cost and time spends. There are several limitations with the present study. First, the heterogeneity among the studies included. Various ultrasonic devices and protocols were employed among the studies, and most importantly, the differences between the treatment periods. Second, the difference of the outcome measurements among the studies, most importantly, the grip strength measurement. The grip strength is an important marker for LELE recovery. The differences in the measurement procedures may result in bias and make the conclusion uncertain. Finally, as non-RCTs were also included, the analysis power and evidence level were weakened. Further well-designed studies should be made to investigate the effect of ultrasound therapy on grip strength recovery.

## 5 Conclusion

The present study demonstrated the ultrasound therapy is helpful to relieve pain for tennis elbow patients, but no such benefit could be found for grip strength. However, the ultrasonic treatment group showed no advantage against other conservative treatments like rest and brace.

## Author contributions

**Conceptualization:** Dongni Luo, Bingyan Liu.

**Data curation:** Dongni Luo, Bingyan Liu.

**Formal analysis:** Dongni Luo, Bingyan Liu, Shengxin Fu.

**Funding acquisition:** Dongni Luo.

**Investigation:** Dongni Luo, Bingyan Liu, Shengxin Fu.

**Methodology:** Dongni Luo, Bingyan Liu, Lini Gao, Shengxin Fu.

**Project administration:** Dongni Luo.

**Resources:** Dongni Luo, Bingyan Liu, Lini Gao, Shengxin Fu.

**Software:** Dongni Luo, Bingyan Liu, Lini Gao.

**Supervision:** Dongni Luo.

**Validation:** Dongni Luo, Bingyan Liu, Lini Gao.

**Visualization:** Dongni Luo.

**Writing – original draft:** Dongni Luo.

**Writing – review & editing:** Dongni Luo.
